# Discovery of Polyphenolic Natural Products as SARS-CoV-2 M^pro^ Inhibitors for COVID-19

**DOI:** 10.3390/ph16020190

**Published:** 2023-01-28

**Authors:** Nadine Krüger, Thales Kronenberger, Hang Xie, Cheila Rocha, Stefan Pöhlmann, Haixia Su, Yechun Xu, Stefan A. Laufer, Thanigaimalai Pillaiyar

**Affiliations:** 1 Infection Biology Unit, German Primate Center, Leibniz Institute for Primate Research Göttingen, Kellnerweg 4, 37077 Göttingen, Germany; 2 Institute of Pharmacy, Pharmaceutical/Medicinal Chemistry and Tübingen Center for Academic Drug Discovery, Eberhard Karls University Tübingen, Auf der Morgenstelle 8, 72076 Tübingen, Germany; 3 Cluster of Excellence iFIT (EXC 2180) “Image-Guided & Functionally Instructed Tumor Therapies”, University of Tübingen, 72076 Tübingen, Germany; 4 School of Chinese Materia Medica, Nanjing University of Chinese Medicine, Nanjing 210023, China; 5 Faculty of Biology and Psychology, Georg-August-University Göttingen, 37073 Göttingen, Germany; 6 State Key Laboratory of Drug Research, Shanghai Institute of Materia Medica, Chinese Academy of Sciences, Shanghai 201203, China

**Keywords:** antivirals, coronavirus, COVID-19, covalent drugs, dynamic light scattering, inhibitors, main protease, natural products

## Abstract

The severe acute respiratory syndrome coronavirus 2 (SARS-CoV-2) has forced the development of direct-acting antiviral drugs due to the coronavirus disease 2019 (COVID-19) pandemic. The main protease of SARS-CoV-2 is a crucial enzyme that breaks down polyproteins synthesized from the viral RNA, making it a validated target for the development of SARS-CoV-2 therapeutics. New chemical phenotypes are frequently discovered in natural goods. In the current study, we used a fluorogenic assay to test a variety of natural products for their ability to inhibit SARS-CoV-2 M^pro^. Several compounds were discovered to inhibit M^pro^ at low micromolar concentrations. It was possible to crystallize robinetin together with SARS-CoV-2 M^pro^, and the X-ray structure revealed covalent interaction with the protease’s catalytic Cys145 site. Selected potent molecules also exhibited antiviral properties without cytotoxicity. Some of these powerful inhibitors might be utilized as lead compounds for future COVID-19 research.

## 1. Introduction

Human coronaviruses such as the Middle East respiratory syndrome coronavirus (MERS-CoV), severe acute respiratory syndrome coronavirus 2 (SARS-CoV-2), and SARS-CoV-1 have emerged since the turn of the twenty-first century, causing three epidemics that have posed serious risks to both public health and the economy [1]. In particular, the ongoing SARS-CoV-2-related pandemic coronavirus disease 2019 (COVID-19) has to date killed about 7 million people and infected approximately 635 million people worldwide and the numbers are still rising. Numerous common molecular characteristics between SARS-CoV-1 and other bat coronaviruses can be found in SARS-CoV-2, a coronavirus from the genus beta-coronavirus [2]. It is highly contagious and fatal for elderly patients with co-morbid conditions [3,4]. A primary objective is the development of a potent antiviral drug, and various viral targets have been considered. Currently, the three antiviral medications approved by the FDA for the treatment of COVID-19 infection are remdesivir and molnupiravir, both targeting the viral RNA-dependent RNA polymerase (RdRp), as well as nirmatrelvir, which targets the SARS-CoV-2 M^pro^. Additionally, a variety of peptidomimetic compounds that successfully inhibit specific SARS-CoV-2 proteases have been identified [5,6,7,8,9], and many medications have been investigated for potential repurposing as COVID-19 treatments [10].

Members of the *Coronaviridae* family, which include seven human coronaviruses, harbor a positive-sense single-stranded RNA genome [11]. Host ribosomes translate the genome of these enveloped viruses into the two polyproteins PP1a and PP1b [1,12]. The main protease, also known as chymotrypsin-like protease or 3C-like protease (3CL^pro^), and a papain-like protease (PL^pro^), cleave the polyproteins to produce mature non-structural functional proteins, such as RdRp and helicase, which are necessary for the completion of the viral replication cycle [13].

In most cases, cleavage of viral proteins by M^pro^ occurs at least 11 times, with substrate specificity exhibited by the efficient cleavage of peptides such as (Leu, Phe, Met, Val)-Gln↓(Ser, Ala, Gly) sequences (the cleavage site is indicated by ↓). It has also been well-established that the substrate-binding sites exhibit an incredibly high degree of conservation, particularly for the critical S1/S2 subsite [14,15,16]. Because of its critical role in processing polyproteins, high degree of conservation, absence of a human analog protease, and involvement in polyprotein processing, M^pro^ is an attractive target for the development of broad-spectrum antiviral drugs. Numerous M^pro^ inhibitors consist of substrate-derived peptides and small molecules with a covalent or noncovalent mechanism of action [11,12,17]. As M^pro^ is one of the most well-characterized therapeutic targets in CoV [5,7,18], finding new M^pro^ inhibitors with various chemical structures is essential to accelerating drug development against highly pathogenic CoVs.

Natural products are an important source of new chemical phenotypes, and the natural phytochemicals that exhibit anti-CoV activity have been extensively summarized [19,20]. Xu et al. recently reported several natural polyphenolic compounds as covalent inhibitors of SARS-CoV-2 M^pro^ and identified pyrogallol as a novel warhead moiety [21,22]. In the present study, we collected several electrophilic natural products and polyphenolic compounds and investigated them for their M^pro^ inhibitory activities. Additionally, the co-crystal structure of SARS-CoV-2 M^pro^ in complex with robinetin was determined, which confirmed the covalent bond formation. Selected active compounds were further tested in Calu-3 cells for antiviral activity.

## 2. Results and Discussion

### 2.1. The M^pro^ Inhibition Assay

The assay was carried out using a newly developed fluorogenic substrate (Dacyl- KTSAVLQSGFRKME-Edans), as previously described [22]. A fluorescence resonance energy transfer (FRET) protease assay was utilized to measure the inhibitory activity of the investigated compounds against the SARS-CoV-2 M^pro^. Each compound was tested at a concentration of 10 µM, and for the compounds that showed >30% inhibition, the half-maximum concentration (IC_50_) was measured (Table 1).

The bicyclic conjugated sesquiterpene ketone (+)-nootkatone, which resembles a grapefruit, is widely utilized in the food, fragrance, cosmetics, and pharmaceutical industries [23,24]. The active ingredient in *Cyperus rotundus*, a well-known oriental traditional medicine, is (+)-nootkatone, which has antiplatelet properties. Additionally, there is proof of its potential effectiveness against *Staphylococcus aureus* biofilms. We selected (+)-nootkatone as a potential candidate as it contains the Michael acceptor in the ring system that might be reactive towards the Cys145 of M^pro^; however, it demonstrated only a moderate inhibition (32.04%).

Oridonin is a bioactive ent-kaurane diterpenoid natural product isolated from *Rabdosia rubescens* that has been widely used in traditional Chinese medicine [25,26]. As reported previously, oridonin possesses anti-tumor, antibacterial, and anti-inflammatory effects. Oridonin has been demonstrated to inhibit NF-B or MAPK activation to reduce the release of proinflammatory cytokines such as tumor necrosis factor (TNF) and interleukin (IL)-6 [27,28,29] that play a crucial role in the process known as cytokine storm, a signal that may result in the death of patients with severe COVID-19. Oridonin’s molecular targets have been identified as the AKT1 and AKT2 inflammasomes, as well as the NLRP3 inflammasome [30]. A recent study revealed that oridonin covalently modifies the NLRP3 inflammasome, targeting the cysteine 279 of NLRP3 [31].

**Figure 1:**
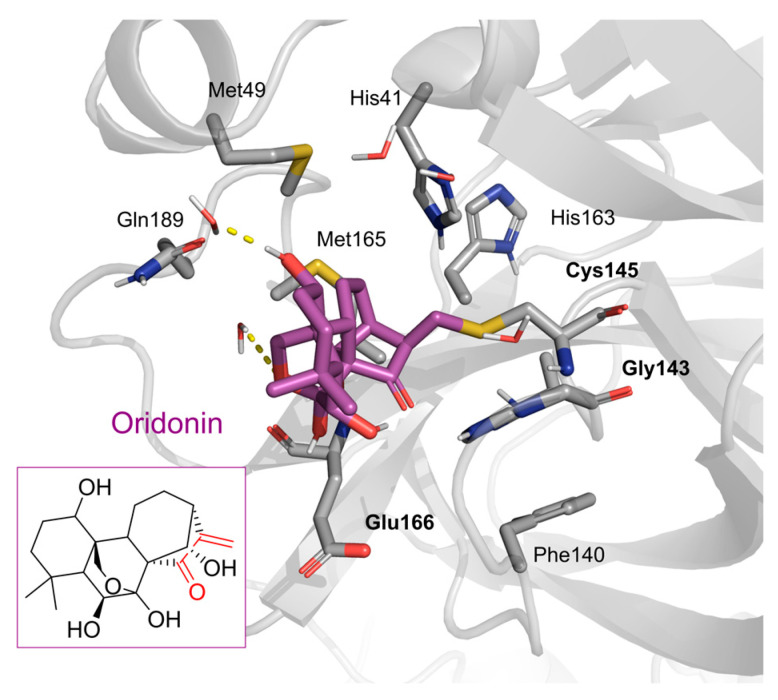
Oridonin-binding mode in the M^pro^ binding site, determined by crystallography (PDB ID: 7VIC). Dashed yellow lines represent polar contacts such as hydrogen bonds and water bridges.

Oridonin has been identified as an inhibitor of M^pro^ with an IC_50_ value of 4.67 µM, which confirmed a previous study showing its M^pro^ inhibitory activity (IC_50_ 2.16 µM [32]). From the literature, it is known that oridonin covalently modifies M^pro^ by targeting Cys145. The M^pro^ crystal structure in complex with oridonin (PDB ID: 7VIC [32], resolution: 2.10 Å, Figure 1) not only confirms the Cys145-covalent bond previously shown by mass spectrometry and kinetic studies but also shows one unique covalent binding mechanism that does not rely on peptidomimetics. Oridonin is stabilized by the classical hydrogen bond interaction with Glu166′s backbone and its hydroxyl group, with the support of hydrophobic interactions with the side-chains of Met49 and Met165, as well as His41 and His163, to some extent.

The xanthone α-mangostin, which was discovered in *G. mangostana*, exhibits a variety of biological effects inhibiting both fatty acid synthase and HIV-1 protease (IC_50_ values of 5.54 and 5.12 µM, respectively). α-Mangostin is effective against methicillin-sensitive and *S. aureus*-resistant strains. It reduces the synthesis of prostaglandin E2 (PGE2, Cay-14010) and nitric oxide (IC_50_ of 12.4 and 11.1 µM, respectively). α-Mangostin exhibited weak M^pro^ inhibition with an IC_50_ value of 25.12 µM.

Robinetin, also known as 5-deoxymyricetin, is a bioactive natural flavonoid. The compound is known for its anti-mutagenesis [33], anti-tumorigenesis [34], and atheroprotective effect [35]. Recent computational work suggests that it could be a potential candidate as a M^pro^ of SAR-CoV-2 inhibitor [36]. In our testing conditions, this compound significantly inhibited M^pro^, with an IC_50_ value of 0.96 µM. The potency of robinetin is almost similar to that of myricetin (IC_50_ 0.649 µM) whose M^pro^ inhibitory activity was confirmed once again in the present study. In contrast, fisetin, which bears catechol in ring C, showed only 11% M^pro^ inhibition, suggesting the presence of pyrogallol to be important for the M^pro^ inhibition.

**Figure 2:**
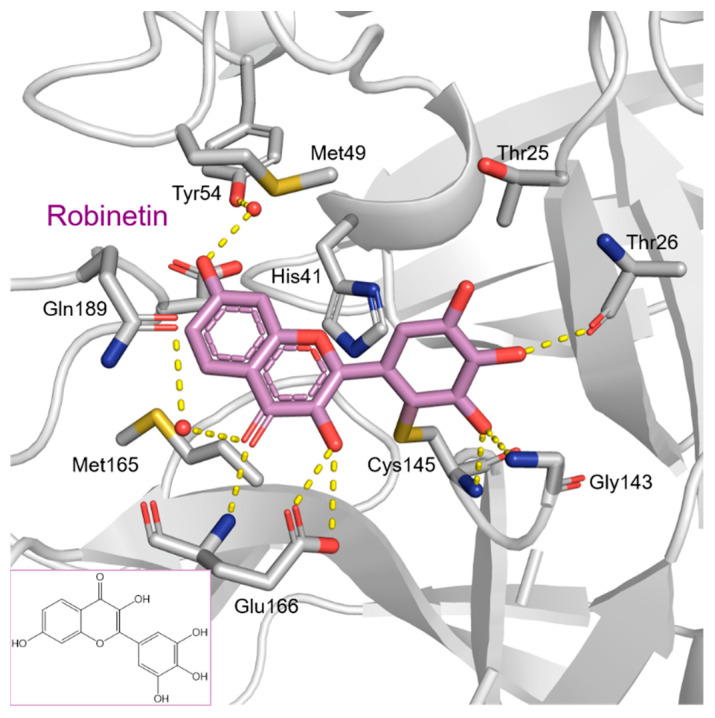
The covalent-binding mode of robinetin in the M^pro^ ligand-binding site revealed by X-ray crystal structure (PDB code: 8HI9). Dashed yellow lines represent polar interactions (hydrogen bonds and water bridges).

To precisely illustrate the binding mode of the inhibitor, a crystal structure of the SARS-CoV-2 M^pro^ in complex with robinetin was determined at 2.28 Å resolution (PDB ID: 8HI9, Figure 2). Robinetin bound in the substrate-binding site at the surface of the protease and mainly occupies the S1 and S2 subsites, similar to the binding mode of myricetin with M^pro^ [21]. At the S1 subsite, the pyrogallol ring establishes a covalent bond with the catalytic residue Cys145. In addition to this covalent binding interaction, the pyrogallol’s hydroxyl group displays hydrogen bonds with the backbone of Gly143/Cys145/Thr26 (part of the oxyanion hole). At the S2 subsite, two hydroxyl groups of the chromone moiety form hydrogen bonds through a water molecule with the side-chain carboxyl group of Glu166 and Tyr54, respectively. Additionally, the carbonyl group of the chromone moiety establishes hydrogen bonds with the main chain of Glu166 and Gln189 through a water molecule. Moreover, robinetin is also stabilized by hydrophobic interactions with the side-chains of Met49/Met165/His41/Thr26. Overall, the crystal structure revealed a covalent binding mode of robinetin to the catalytic site of the SARS-CoV-2 M^pro^ using the pyrogallol ring as a reaction warhead.

Myricetin, a naturally occurring flavonol found in edible plants, has antimutagenic and anticarcinogenic properties. Myricetin inhibits M^pro^ with an IC_50_ value of 0.645 µM at a concentration of 10 µM. Recently, Xu. et al. discovered myricetin to have M^pro^ inhibitory action [21]. The molecule’s binding mode demonstrated that the pyrogallol group functions as an electrophile to covalently alter the catalytic cysteine.

**Figure 3:**
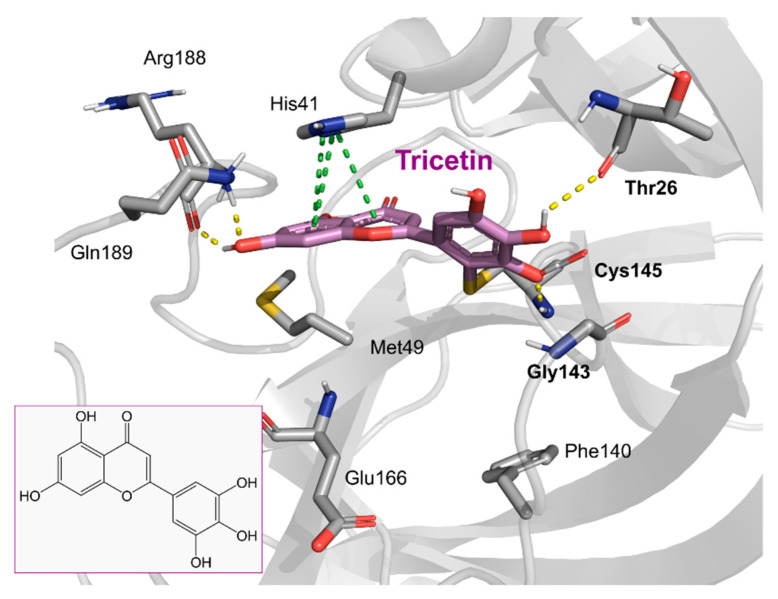
The putative tricetin binding mode in the M^pro^ binding site. Dashes represent different interaction types: green—π-stacking, yellow—polar contacts such as hydrogen bonds.

Tricetin activates the Nrf2/HO-1 signaling pathway, protecting against 6-OHDA-induced neurotoxicity in the Parkinson’s disease model. It effectively inhibits the protein–protein interaction of Keap1–Nrf2 [37]. It had a weaker inhibitory effect on M^pro^ than its 3-OH derivative, myricetin, with an IC_50_ value of 7.84 µM. It is possible that the 3-OH in myricetin or robinetin facilitates hydrogen bonding with the M^pro^ active site residues, such as Glu166 (see Figure 2), or that it changes the electronic properties of the ring, facilitating π-mediated interactions with His41 (Figure 2 and Figure 3). Tricetin’s putative binding mode (Figure 3) suggests a similar binding mode to its derivatives myricetin and robinetin, resulting in covalent interaction between the pyrogallol ring and Cys145. The oxygen in the pyrone ring forms an H-bond interaction with His41, and the catechol hydroxyls form H-bond interactions with Gln189 and Arg188, respectively.

Another flavonoid, luteoline, a potent Nrf2 inhibitor, was unable to inhibit M^pro^ at the concentration of 10 µM. Interestingly, scutellarein, the main bioactive component extracted from *Erigeron breviscapus*, inhibited M^pro^ with moderate potency and an IC_50_ value of 6.07 µM, confirming previous findings [38,39]. Despite containing the pyrogallol unit in its core structure, the natural product myricitrin, the 3-O-L-rhamnopyranoside of myricetin, did not inhibit M^pro^. It suggested that the 3-O-L-rhamnopyranoside might have steric clashes which inhibit its ability to interact with the enzyme. Quercitrin was also unable to inhibit the M^pro^ enzyme.

**Figure 4:**
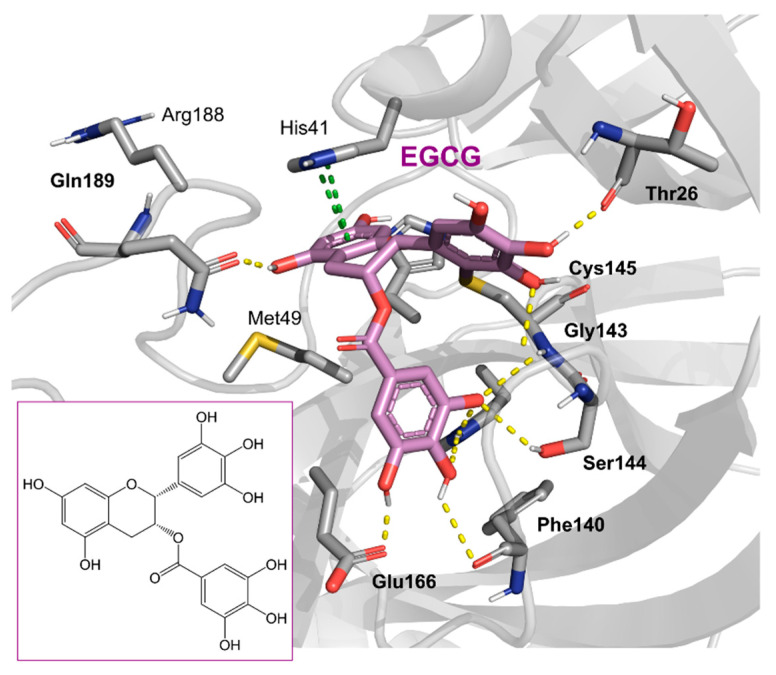
Potential binding mode of EGCG in the M^pro^ active site. Dashes represent different interaction types: green—π-stacking, yellow—polar contacts such as hydrogen bonds.

Chinese herbal medicine contains the compound EGCG (CHM). The tea leaf, especially green tea, is a rich source of EGCG [40]. Just a few of the viruses that EGCG has proven to be effective against include adenovirus, influenza, Zika, herpesvirus, and hepatitis virus [41]. Zuo et al., in particular, showed that EGCG has an inhibitory effect on the NS3 serine protease of the hepatitis C virus (HCV) [42]. EGCG was found to inhibit SARS-CoV-2 M^pro^ in in vitro studies, with an IC_50_ value of 73 µM [43]. It was also recently found to inhibit SARS-CoV-2 M^pro^ with IC_50_ values ranging from 0.874 to 4.24 µM [44,45]. In our investigation, EGCG was able to completely inhibit the SARS-CoV-2 M^pro^ activity (IC_50_ value of 0.228 µM). Interestingly, the host suffers significant damage from proinflammatory cytokines that are overproduced [46]. The need for drugs that can treat COVID-19 hyperinflammation is therefore very high in the clinical and research communities. In this regard, EGCG may be a promising therapy for COVID-19 to reverse hyperinflammation. Previous research has shown that EGCG can effectively block the JAK/STAT pathway, which controls the production and release of a number of cytokines and chemokines [47,48]. Additionally, EGCG has been shown to inhibit the canonical NF-κB pathway [49,50] which is crucial for controlling the expression of proinflammatory cytokines such as IL-1, TNF-α, IL-8, and IL-6. Both COVID-19 and cytokine storm syndrome generate all of these cytokines. Due to its low toxicity and potent intestinal absorption, EGCG also has additional benefits [51]. The proposed binding mode of EGCG relies on the covalent binding to Cys145, in a similar manner to robinetin. The benzenetriol moiety establishes multiple hydrogen bond interactions with the backbone atoms of Gly143, Cys145 and the side-chain of Ser144 in the S1 subsite (Figure 4). The second benzenetriol moiety occupies the pocket between Glu166 and Phe140, establishing hydrogen bond interactions with those parts. Lastly, the chromone moiety interacts with Gln189 and is stabilized by stacking interactions with His41.

**Figure 5:**
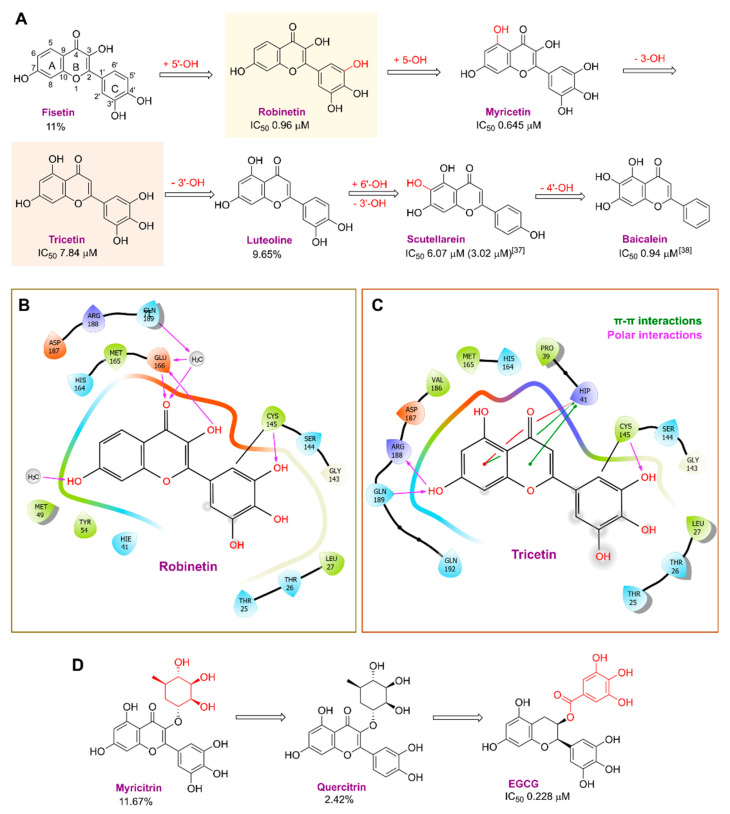
The structure–activity relationships of polyphenolic compounds. The introduction of 5′-OH (Robinetin) and 5-OH (Myricetin) (**A**); an exemplary binding mode for robinetin derived from the crystal structure (**B**); and the docking model for tricetin (**C**); as well as 2D representations of the polyphenols coupled at the 3′ subunit (**D**).

Figure 5 summarizes the structure–activity relationships of these polyphenolic compounds in relation to the SARS-CoV-2 M^pro^. In general, it was found that the trihydroxyl groups (pyrogallol) in either ring A or B were crucial for the M^pro^ inhibitory activity. This outcome confirms the earlier discovery that pyrogallol is a novel covalent warhead group for the development of SARS-CoV-2 M^pro^ inhibitors [21]. Myricitrin did not, however, inhibit M^pro^, even though its core structure includes a pyrogallol unit (Figure 5D). This suggested that the 3-*O*-*α*-l-rhamnopyranoside might have steric clashes in its interaction with the enzyme. It was discovered that the molecule’s catechol unit had no effect on M^pro^ activity (compare fisetin vs. robinetin; luteoline vs. tricetin). The M^pro^ inhibitory activity of robitenin (Figure 5A,B) is favored by the presence of a hydroxyl group at position 3 on ring B of the molecule, leading to the interaction with Glu166. This interaction is absent in tricetin (Figure 5A,C), which could explain the reduced binding activity. On the other hand, it was revealed that the presence of a 4-hydroxy group in ring C decreased inhibitory activity when compared to scutellarein and baicalein. In the current study, EGCG was discovered to be the most potent M^pro^ inhibitor (Figure 5D).

**Figure 6:**
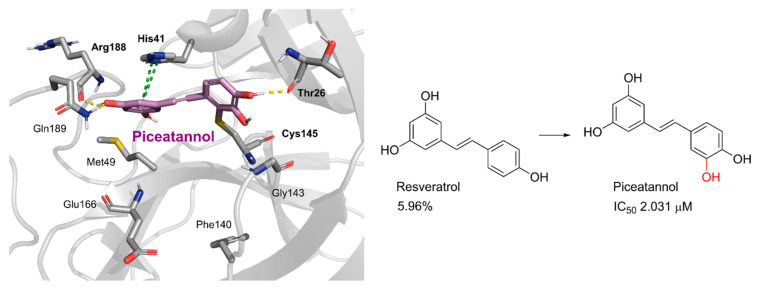
Docking study of piceatannol in the M^pro^ binding site. Dashes represent different interaction types: green—π-stacking, yellow—polar contacts such as hydrogen bonds.

Piceatannol is a resveratrol metabolite and a bioactive stilbenoid of the catechol type that is present in both mycorrhizal and non-mycorrhizal roots of Norway spruces (*Picea abies*) [52]. Additionally, it is present in the seeds of the palm. The protein kinase A subunit, pKC, and MLC are all reversibly inhibited by this cell-permeable substrate. Piceatannol inhibited LMP2A [53], a viral protein-tyrosine kinase thought to be involved in leukemia, non-lymphoma, Hodgkin’s disease, and other illnesses connected to the Epstein-Barr virus. Piceatannol inhibits the M^pro^ with an IC_50_ value of 2.031 µM. However, its parent molecule, resveratrol, was unable to inhibit the enzyme. The molecular docking study suggests that the catechol unit in piceatannol is crucial for its inhibitory activity as one of the catechol rings interacts with the P1 pocket (Cys145, Figure 6) of M^pro^, which is missing in the case of resveratrol. Meanwhile, the resorcinol ring stabilizes π–π interactions with His41 and further polar contacts with Gln189.

**Figure 7:**
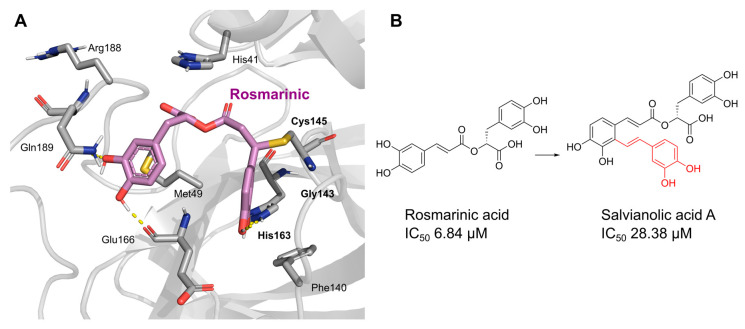
Rosmarinic potential binding mode within the M^pro^ catalytic binding site (**A**). Dashed yellow lines represent polar contacts such as hydrogen bonds. The introduction of 3,4-dihydroxy phenyl acrylate in rosmarinic acid led to salvianolic acid, which reduced the activity (**B**).

Rosmarinic acid, a naturally occurring substance obtained from the rosemary plant (*Rosmarinus officinalis*) [54,55], is chemically the ester of caffeic acid with 3,4-dihydroxyphenyl)-lactic acid. This substance has been shown to have antiviral, antibacterial, and anti-inflammatory effects [56]. Because of this, it is utilized in a number of lemon balm remedies [57] as well as several ointments for sports injuries. The formation of orthoquinones by oxidation of the phenolic hydroxyl groups in rosmarinic acid is well recognized. These associate with peptides and subsequently render them inactive [58]. In our work, rosmarinic acid inhibited M^pro^ with an IC_50_ value of 6.84 µM. We propose that it might covalently alter the M^pro^ interactions between Cys145 and the Michael acceptor donor, instead of orthoquinone generated by oxidizing the phenolic hydroxyl groups of rosmarinic acid, as originally proposed by other groups. Covalently bound rosmarinic acid frees the hydroxyl groups to interact with His163 and the Glu166 backbone (Figure 7).

**Figure 8:**
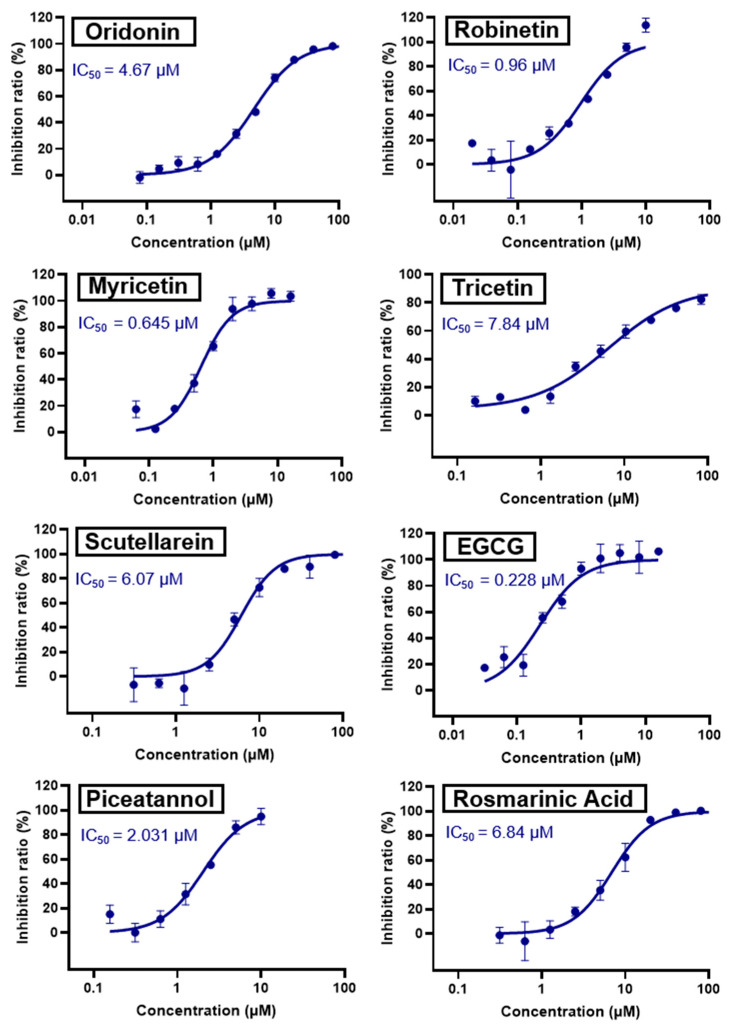
Concentration-dependent inhibition of SARS-CoV-2 M^pro^ by the best inhibitors of the present series: Oridonin, robinetin, myricetin, tricetin, scutellarein, L-epigallocatechin gallate, piceatannol, rosmarinic acid.

Salvianolic acid A (SA), also known as Dan Phenolic Acid A, is a water-soluble chemical that is derived from Radix Salvia miltiorrhiza (Danshen). It has antioxidant and free radical-scavenging properties, and it also prevents protein–protein interactions that are carried out by the SH2 domains of the Src-family kinases Src and Lck. SA stops the progression of diabetes in diabetic rats fed a high-fat diet and the development of fibrosis caused by streptozotocin. Hepatic triglyceride (TG) levels are significantly decreased while hyperlipidemia is improved by an oral dose of SA at 0.3 mg/kg twice daily for 16 weeks. Salvianolic acid A is being studied in the NCT03908242 clinical trial (Phase I Study of Continuous Administration of Salvianolic Acid A Tablets). This substance inhibited M^pro^ significantly less effectively than its analog rosmarinic acid, with an IC_50_ value of 28.38 µM, which suggests that the extra 3,4-dihydroxy phenyl acrylate unit in salvianolic acid may have caused steric hindrance in binding at the M^pro^ active site. The concentration−inhibition curves for selected compounds are shown in Figure 8.

### 2.2. Cytotoxicity and Anti-SARS-CoV-2 Activity

Prior to examining the antiviral activity in Calu-3 cells, selected M^pro^ natural product inhibitors were tested in the cell line at a high concentration of 10 µM for cytotoxicity. No tested substance exhibited cytotoxicity, as shown in Figure S1.

The most common method for determining antiviral efficacy against respiratory pathogens in vitro uses the human lung-derived Calu-3 cells, in which SARS-CoV-2 enters the cells in a TMPRSS2-dependent manner. To assess each of the chosen M^pro^ inhibitors, Calu-3 cells were infected with SARS-CoV-2.

Cells were incubated with 10-fold serial dilutions of each inhibitor or DMSO (solvent control) for 1 hour prior to infection and for 24 h after infection (p.i.) with the SARS-CoV-2 isolate NK, Pango lineage B.1.513 (kindly provided by Stephan Ludwig, Institute of Virology, University of Münster, Germany) at a multiplicity of infection (MOI) of 0.01. Cell culture supernatants were harvested at 24 h p.i. and titrated on Vero E6 cells to determine viral titers, given as plaque-forming units (PFU) per milliliter (Figure S2). The well-known SARS-CoV-2 inhibitor molnupiravir served as the positive control.

**Figure 9:**
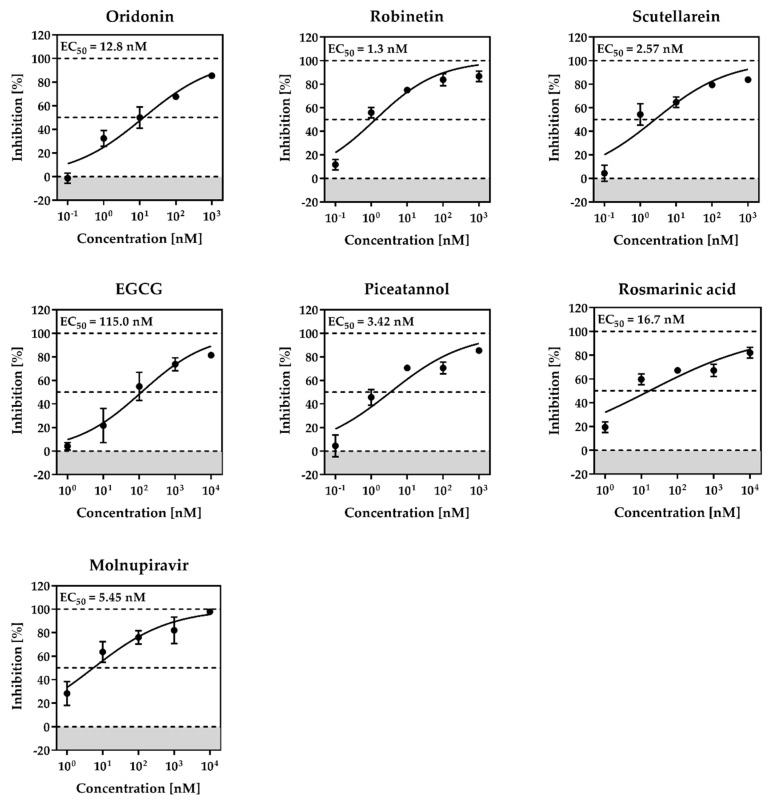
Antiviral activity and cytotoxicity of robinetin, piceatannol, rosmarinic acid, scutellarein, EGCG, oridonin in Calu-3 cells. Calu-3 cells were incubated with 10-fold serial dilutions (10−0.001 or 1-0.0001 μM) of each inhibitor or DMSO (solvent control) for 1 h followed by infection with SARS-CoV-2 at a multiplicity of infection (MOI) of 0.01. After virus inoculation, cells were further incubated with the respective inhibitors for 24 h. Supernatants were harvested, and viral titers were determined by titration on Vero E6 cells. For normalization, viral titers of DMSO-treated cells were set as 0% inhibition. Means ± SDs from three biological replicates are presented.

Based on these findings, concentration-dependent inhibition curves were generated for all active inhibitors to determine EC_50_ values (Figure 9). All tested compounds showed inhibition of viral titers above 80% at a concentration of 10 µM (robinetin, 89.6%; piceatannol, 81.0%; rosmarinic acid, 81.0%; scutellarein, 87.0%; EGCG, 81.4; oridonin, 89%).

EC_50_ values ranged between 1.3 nM (robinetin) and 115 nM (EGCG, Figure 9), which were several-fold more potent compared to their M^pro^ inhibitory activities. These natural products have been reported for several targets, and the inhibition of virus replication in cells might be contributed by the inhibition of non-specific targets as well. Therefore, these inhibitors should be used with caution.

### 2.3. Colloidal Aggregation Assays

In early drug discovery, aggregation is a common reason for false positives when organic [59], drug-like molecules are added to aqueous media and spontaneously form colloidal particles [59,60,61,62]. Because proteins are trapped on the colloid surface and partially unfolded there [63], the resulting liquid particles are tightly packed spheres that promiscuously inhibit proteins [64,65]. Due to enzyme crowding on the particle’s surface [65], the resulting inhibition is reversible by disrupting the colloid and is characterized by an incubation effect lasting several minutes. Colloids can frequently be broken up by adding non-ionic detergents like Triton X-100 in small amounts, frequently at sub-critical micelle concentrations [66]. Therefore, one frequent perturbation to quickly identify aggregates is to add detergent to counter screens against model enzymes such as AmpC β-lactamase or malate dehydrogenase (MDH). Nuclear magnetic resonance (NMR) and dynamic light scattering (DLS) are two well-suited biophysical techniques that can detect aggregation [67], especially with DLS, as the colloids typically form particles in the 50 to 500 nm radius size range.

The Shoichet group recently reported that colloidal aggregation can cause false positive results in studies to repurpose drugs to target SARS-CoV-2. We chose ECGC and robinetin, two potent natural products, for the current study to find out whether they could form colloidal particles. Each substance was initially screened against MDH in triplicate at 31.6 µM. Compounds that demonstrated > 40% enzyme inhibition against MDH were tested in triplicate for detergent reversibility at 31.6 µM with or without 0.01% (*v*/*v*) Triton X-100. As previously described, enzymatic reactions were carried out and observed. Each compound was screened at 31.6 µM by DLS and the critical aggregation concentration (CAC) data for each compound was determined if colloidal particle formation was detected.

**Figure 10:**
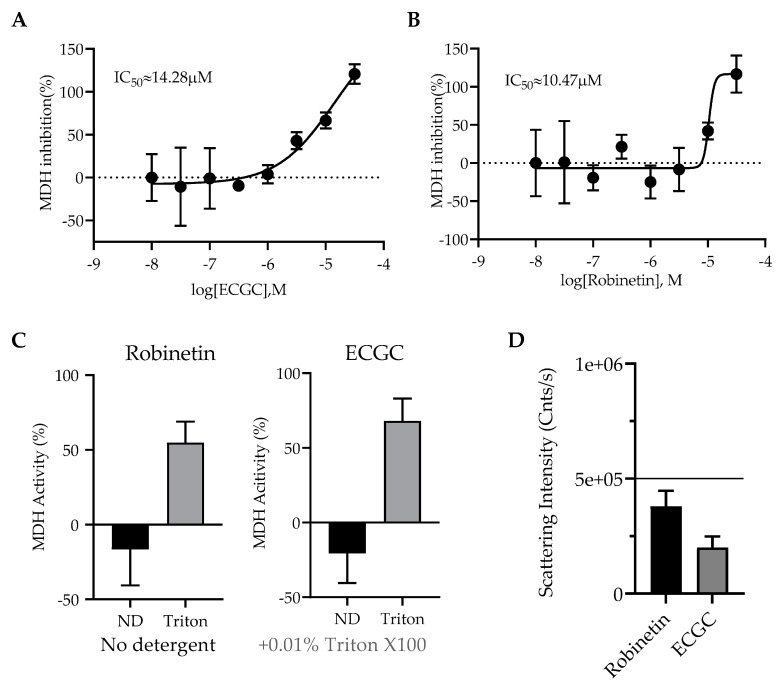
Aggregation assays. Malate dehydrogenase (MDH) enzymatic screen for ECGC (**A**) and robinetin (**B**). (**C**) Malate dehydrogenase (MDH) enzymatic activity with or without 0.01% Triton X-100 at 31.6 µM. (**D**) Scattering intensity of compounds ECGC and robinetin that do not form colloidal-like particles measured by DLS at 31.6 µM.

The outcomes demonstrated that both EGCG and robinetin had a weak inhibition of MDH with IC_50_ values of 14.28 and 10.47 µM, respectively (Figure 10A,B), and these inhibitions were reversed in the presence of detergent (Figure 10C). However, neither of these compounds produced colloidal-like particles as detected by DLS (Figure 10D) indicating that they can both be categorized as non-specific inhibitors and should be used with caution at concentrations where MDH inhibition was shown.

## 3. Materials and Methods

### 3.1. Compounds

All compounds are >95% pure by HPLC, as reported by the vendors. Compounds were ordered from Sigma-Aldrich, AmBeed, or TCI Europe.

### 3.2. The Inhibition Assay of SARS-CoV-2 M^pro^

The expression and purification of recombinant SARS-CoV-2 M^pro^ were performed as previously reported [22]. A fluorescence resonance energy transfer (FRET) protease assay was applied to measure the inhibitory activity of compounds against the SARS-CoV-2 M^pro^. The fluorogenic substrate (Dacyl-KTSAVLQSGFRKME-Edans) was synthesized by GenScript (Nanjing, China). The FRET-based protease assay was performed as follows. SARS-CoV-2 M^pro^ (50 nM) was mixed with compounds in assay buffer (50 mM Tris-HCl, pH 7.3, 1 mM EDTA) and incubated for 10 min. The reaction was initiated by adding fluorogenic substrate at a final concentration of 10 µM. Then, the fluorescence signal at 340 nm (excitation)/490 nm (emission) was measured every 1 min for 10 min using a Bio-Tek SynergyH1 plate reader. The initial velocities of reactions with compounds added at various concentrations compared to the reaction with DMSO added were calculated and used to generate inhibitory profile curves for IC_50_ determination.

### 3.3. Dynamic Light Scattering (DLS)

A filtered 50 mM KPi buffer with a pH of 7.0 was used to create the samples, and the final DMSO concentration was set at 1% (*v*/*v*). The detection of colloidal particle generation was carried out using DynaPro Plate Reader II (Wyatt Technologies, Promega, Madison, WI, USA). Each substance was examined three times at 31.6 µM. The 8-point half-log dilutions of the compounds were performed in triplicate if colloids were discovered by DLS. To determine the critical aggregation concentration (CAC), data for each compound were spilt into two data sets based on aggregating (i.e., >10^6^ scattering intensity) and non-aggregating (i.e., <10^6^ scattering intensity) and fitted with separate nonlinear regression curves, and the point of intersection was determined using GraphPad Prism software version 9.1.1 (San Diego, CA, USA).

### 3.4. Malate Dehydrogenase Inhibition Assays

Using a CLARIOstar Plate Reader at room temperature, enzyme inhibition tests were performed (BMG Labtech, Ortenberg, Germany). The final concentration of 1% (*v*/*v*) DMSO was used to prepare samples in 50 mM KPi buffer at pH 7.0. An amount of 2 nM malate dehydrogenase (MDH) was added to the compounds and incubated for 5 min. In order to initiate the MDH reactions, 200 M each of oxaloacetic acid (32,4427, Sigma Aldrich) and nicotinamide adenine dinucleotide (NADH) (54,839, Sigma Aldrich, Saint Louis, MO, USA) were added. The change in absorbance was observed at 340 nm over a period of 1 min and 30 s. To calculate the percentage of enzyme activity, initial rates were divided by the DMSO control rate. At 31.6 µM, each compound was examined in triplicate. Version 9.1.1 of the GraphPad Prism program was used to analyze the data (San Diego, CA).

Compounds that inhibited MDH by > 40% were tested in triplicate at 31.6 µM with or without 0.01% (*v*/*v*) Triton X-100 to determine whether they were reversible detergents. As previously mentioned, enzymatic reactions were conducted and monitored.

### 3.5. Cytotoxicity and Antiviral Assays

Cell Viability Assay. To exclude cytotoxic effects, the viability of Calu-3 cells treated with inhibitors was determined using the CellTiter-Glo Luminescent Cell Viability Assay Kit (Promega, Madison, WI, USA) according to the manufacturer’s protocol. Briefly, Calu-3 cells were grown in 96-well plates until they reached 50−60% confluency. Next, cell culture supernatants were removed and cells were incubated with cell culture medium containing 0.1% of DMSO (solvent control) or 10 µM of the respective M^pro^ inhibitors for 24 h. After removal of the cell culture supernatants, 50 μL of the CellTiter-Glo substrate was added to each well followed by incubation on a rocking platform for 30 min. Finally, samples were transferred into white 96-well plates, and luminescence was measured using a Hidex Sense plate luminometer (Hidex).

Antiviral Activity. All work with infectious SARS-CoV-2 was conducted under BSL-3 conditions at the German Primate Centre, Göttingen, Germany. Calu-3 cells were grown in 48-well plates. After reaching ~70% confluency, the cell culture medium was removed and cells were incubated with 0.1% DMSO (control) or 10-fold serial dilutions (10−0.001 or 1–0.0001 μM) of M^pro^ inhibitors for 1 h at 37 °C. Next, the cell culture supernatants were removed, and cells were infected with SARS-CoV-2, Pango lineage B.1.513, at an MOI of 0.01 for 1 h at 37 °C. The virus inoculum was removed, cells were washed with PBS three times and further incubated in a cell culture medium containing the respective inhibitor or DMSO for 24 h. Virus-containing cell culture supernatants were harvested at 1 d post infection (p.i.) and stored at −80 °C until further usage. Serial dilutions of virus-containing supernatants were titrated on Vero E6 cells followed by methylcellulose overlay to determine viral titers.

### 3.6. Molecular Modelling

Using LigPrep (implemented in Maestro 2021v4), three-dimensional ligand structures were created, tautomers and diastereoisomers were produced [68], and Epik was used to predict the protonation of the compounds in the pH range of 7.0 to 2.0 [69]. The SARS-CoV-2 M^pro^ protein structure was previously prepared from the PDB 7DPP, using the Protein Wizard Preparation tool with standard options. Covalent docking was performed using CovDock [70], using the Cys145 as anchor, nucleophilic addition to double bond or Michael addition as reaction types, and generating up to 10 poses for each ligand; the best scored pose was selected and figures were generated using PyMOL 2.5.2.

### 3.7. X-ray Protein Crystallography Experiments

In order to know the exact binding mode of robinetin with the protease, a crystallography study was carried out. As mentioned above, the expression and purification of the recombinant SARS-CoV-2 M^pro^ follow previous protocols [22]. The resulting protein sample was first concentrated to 9 mg/mL and then incubated with 1 mM robinetin to generate the complex sample. Crystallization condition screening was performed at 20 ℃ using a hanging drop vapor-diffusion method by mixing equal volumes (1:1 μL) of the M^pro^–robinetin mixture and reservoir solution. The resulting crystals were flash-frozen in liquid nitrogen with the reservoir solution supplemented with 20% glycerol. X-ray diffraction data were collected at beamline BL19U1 at the Shanghai Synchrotron Radiation Facility [71]. The crystal grown under the conditions of 10–25% PEG6000, 100 mM MES, pH 5.5–6.75, and 3% DMSO resulted in the high-resolution diffraction data and thus was used to solve the complex structure. Molecular replacement using the program PHASER [72] with a search model of PDB code 6M2N was applied to generate the initial model of the complex. The model was further built using Coot [73] and refined with XYZ (reciprocal-space), Individual B factors, TLS parameters, and occupancies implemented in the program PHENIX [74]. The refined structure with a PDB code of 8HI9 was deposited in the Protein Data Bank. The complete statistics and the quality of the solved structure are also shown in Table 2.

## 4. Conclusions

New chemical phenotypes are commonly found in natural goods. In the current work, we evaluated 31 natural compounds and their derivatives as potential SARS-CoV-2 M^pro^ inhibitors. Ten of them were shown to be M^pro^ inhibitors. Robinetin, rosmarinic acid, and its derivative salvianolic acid A were shown to be novel M^pro^ ligands, and the M^pro^ inhibitory effect was reinforced for myricetin, oridonin, scutellarein, and L-epigallocatechin gallate. Utilizing molecular docking studies, the binding mode of potent M^pro^ inhibitors was investigated. Additionally, the structural determination of robinetin with M^pro^ provides a rationale for new analog designs. The strong antiviral activity of the selected potent compounds, especially robinetin with an EC_50_ of 1.3 nM, indicates promising options for further research into these compounds as an antiviral therapy for COVID-19.

## Figures and Tables

**Table 1:** Inhibition of SARS-CoV-2 M^pro^ by natural and synthesized compounds.

Chemical Structure	SARS-CoV-2 M^pro^	
	% Inhibition at 10 μM	IC_50_ (μM)
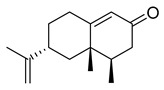 (+)-Nootkaton	32.04	>10
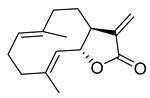 Costunolide	27.96	>10
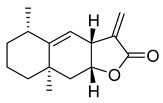 Alantolacton	27.55	>10
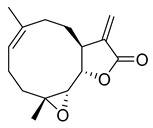 (−)-Parthenolide	20.06	>10
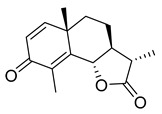 (−)-α-Santonin	24.13	>10
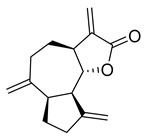 Dehydrocostus lactone	13.39	>10
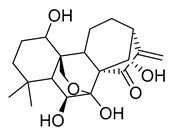 Oridonin	81.87	4.67
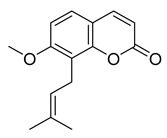 Osthole	4.98	>10
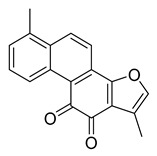 Tanshinone I	7.03	>10
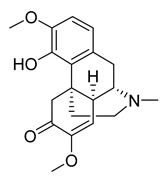 Sinomenine	4.80	>10
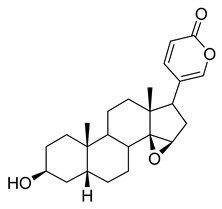 Resibufogenin	6.32	>10
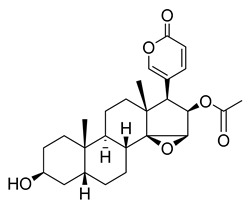 Cinobufagin	0.53	>10
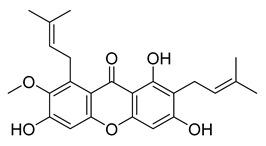 α-Mangostin	38.56	25.12
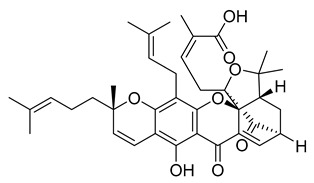 Gambogic acid	22.75	>10
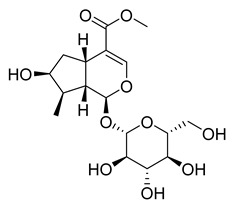 Loganin	29.43	>10
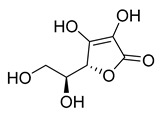 l-Ascorbic acid	22.25	>10
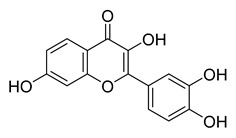 Fisetin	−11.21	>10
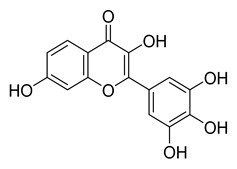 Robinetin	86.13	0.96
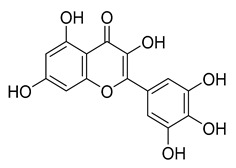 Myricetin	104.00	0.645
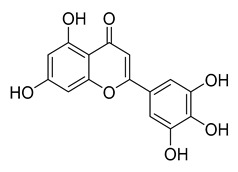 Tricetin	72.28	7.84
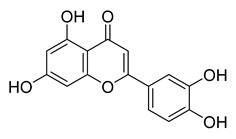 Luteoline	9.65	>10
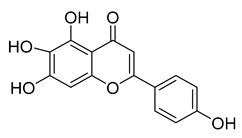 Scutellarein	76.07	6.07
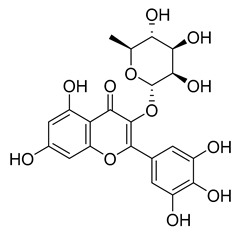 Myricitrin	11.67	>10
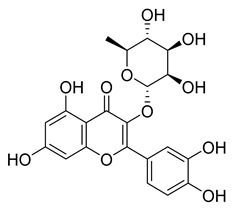 Quercitrin	2.42	>10
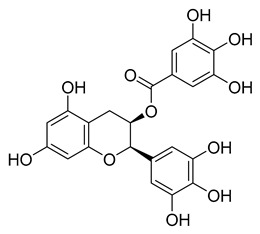 l-Epigallocatechin gallate	105.40	0.228
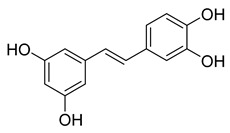 Piceatannol	76.90	2.031
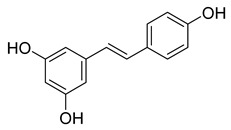 Resveratrol	5.96	>10
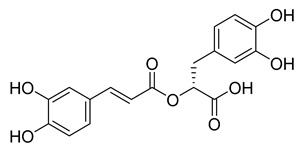 Rosmarinic acid	50.56	6.84
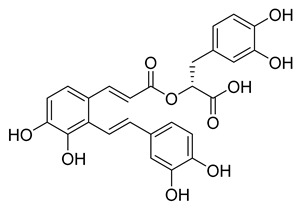 Salvianolic acid A	50.84	28.38
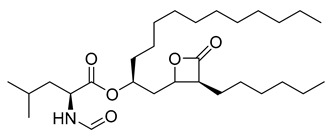 Orlistat	20.99	>10
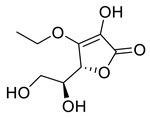 3-*O*-Ethyl-l-ascorbic acid	24.94	>10

**Table 2:** Crystallography data collection and refinement statistics.

	SARS-CoV-2 M^pro^–Robinetin
PDB ID	8HI9
Space Group	P 21
Cell Dimension: a (Å)	44.156
b (Å)	54.182
c (Å)	115.416
Wavelength (Å)	0.979
Reflections (unique)	24333
Resolution Range (Å)	2.28-37.78
Highest-Resolution Shell (Å)	2.28-2.39
Redundancy	6.1(6.2)
I/σ (I)	14.8(6.9)
Completeness (%)	99.0(99.0)
Rwork/Rfree	0.2457/0.2553
Clashscore	1.72
MolProbity Score	0.93
RMS Values	
Bond Length (Å)	0.003
Bond Angle (°)	0.638
Number of Non-hydrogen Atoms	
Protein	4449
Inhibitor	44
Water Oxygen	108
Others	0
B-factor (Å^2^)	
Protein	33.33
Inhibitor	35.13
Water Oxygen	29.42
Ramachandran Plot	
Favored (%)	98.32
Allowed (%)	1.52
Outliers (%)	0.17

## Data Availability

Data is contained within the article and Supplementary Materials. All PDB structures of the proposed binding modes have been made available.

## Supplementary Materials

The following supporting information can be downloaded at: https://www.mdpi.com/article/10.3390/ph16020190/s1. Figure S1. Cell viability of Calu-3 cells treated with M^pro^ inhibitors; Figure S2. Antiviral activity of M^pro^ inhibitors on SARS-CoV-2 infectivity in Calu-3 cells. All PDB structures of the proposed binding modes have been made available.
