# Supplementary material for: Discovery of Polyphenolic Natural Products as SARS-CoV-2 Mpro Inhibitors for COVID-19
Source: Pharmaceuticals (Basel). 2023 Jan 28;16(2):190. doi: 10.3390/ph16020190 (PMC9959258; doi:10.3390/ph16020190)
Supplement: Supplementary file 1 [file pharmaceuticals-16-00190-s001.zip › pharmaceuticals-2086846-supplementary.pdf]

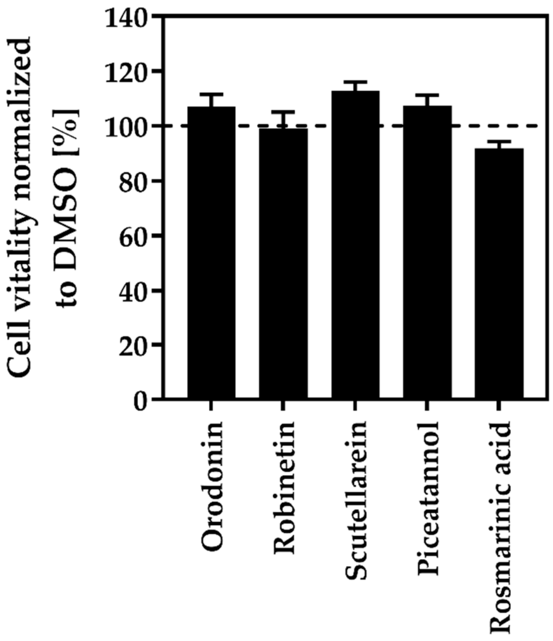

Figure S1. Cell vitality of Calu-3 cells treated with M^{pro} inhibitors. Cells were incubated with 10 µM of each inhibitor or DMSO (solvent control) for 24 h, before cell vitality was determined by luminescent ATP measurement. Values observed for each inhibitor were normalized to luminescence values measured for DMSO treated cells (set as 100%). The graph shows means and SEM of four (Calu-3) independent experiments. Each individual experiments measured as triplicates.

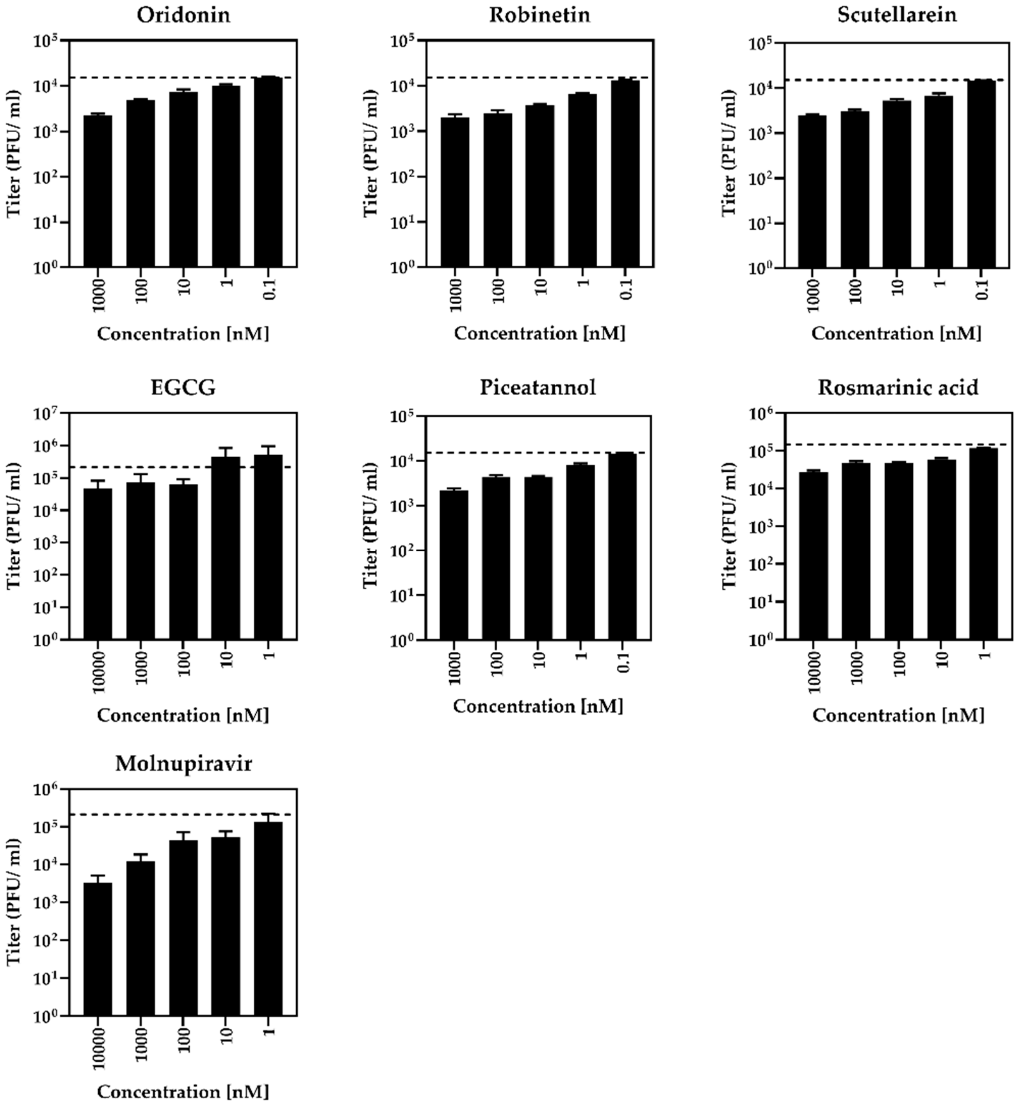

Figure S2. Antiviral activity of M^{pro} inhibitors on SARS-CoV-2 infectivity in Calu-3 cells. Lung-derived human Calu-3 cells were incubated with 10-fold serial dilutions of each inhibitor or DMSO (solvent control) for 24 h, followed by infection with SARS-CoV-2 at an MOI of 0.01. After virus inoculation, cells were further incubated with the respective inhibitors for 24 h. Supernatants were harvested and viral titers were determined by titration on Vero E6 cells and are given as plaque-forming units (PFU)/mL. The graph shows the mean and SD of three independent experiments. Dashed lines indicate the mean titer of SARS-CoV-2 in DMSOtreated Calu-3 cells.
